# Supplementary material for: Incidence of lower limb deep vein thrombosis in patients with COVID-19 pneumonia through different waves of SARS-CoV-2 pandemic: A multicenter prospective study
Source: PLoS One. 2023 Feb 2;18(2):e0280247. doi: 10.1371/journal.pone.0280247 (PMC9894444; doi:10.1371/journal.pone.0280247)
Supplement: Attachment — Submitted filename: Rebuttal to PlosOne.docx [file pone.0280247.s001.docx]

Dear Editor, we are grateful for the opportunity to resubmit our paper entitled “**Incidence of lower limb deep vein thrombosis in patients with COVID-19 pneumonia through different *waves* of SARS-CoV-2 pandemic: a multicenter prospective study.**”

Below you will find the complete rebuttal to the comments and queries posed by the reviewers.

We hope you will now find the manuscript suitable for consideration of publication in your Journal.

Filippo Pieralli and Fulvio Pomero on the behalf of all coauthors.

Journal requirements.

*We noticed you have some minor occurrence of overlapping text with the following previous publication(s), which needs to be addressed:*

*Pieralli F, Pomero F, Giampieri M, Marcucci R, Prisco D, Luise F, et al. (2021) Incidence of deep vein thrombosis through an ultrasound surveillance protocol in patients with COVID-19 pneumonia in non-ICU setting: A multicenter prospective study. PLoS ONE 16(5): e0251966.* [*https://doi.org/10.1371/journal.pone.0251966*](https://doi.org/10.1371/journal.pone.0251966)

*In your revision ensure you cite all your sources (including your own works), and quote or rephrase any duplicated text outside the methods section. Further consideration is dependent on these concerns being addressed.*

The overlapping text with our previous work, *Pieralli F, Pomero F, Giampieri M, Marcucci R, Prisco D, Luise F, et al. (2021) Incidence of deep vein thrombosis through an ultrasound surveillance protocol in patients with COVID-19 pneumonia in non-ICU setting: A multicenter prospective study. PLoS ONE 16(5): e0251966.* [*https://doi.org/10.1371/journal.pone.0251966*](https://doi.org/10.1371/journal.pone.0251966), has been rephrased weather needed.

*Please provide additional details regarding participant consent. In the ethics statement in the Methods and online submission information, please ensure that you have specified what type you obtained (for instance, written or verbal, and if verbal, how it was documented and witnessed). If your study included minors, state whether you obtained consent from parents or guardians. If the need for consent was waived by the ethics committee, please include this information.*

The study protocol was approved by the ethics committee of the coordinating center, Azienda Ospedaliera Universitaria Careggi, Florence, Italy (COCORA protocol 17104). Informed consent was not needed, since point-of-care ultrasonography is a standard of care for the evaluation and monitoring of patients with COVID-19; only signed consent for personal data collection and treatment was requested; whenever the collection of the written consent for personal data collection was not feasible, a verbal consent witnessed by healthcare bystanders was obtained. All data were collected and analyzed anonymously.

*Thank you for stating the following financial disclosure:*

*“NO - No authors received fundings for this work”.*

*The following statement has been now included at the end of the text.*

**None of the authors received specific funding for this work.**

*In your Data Availability statement, you have not specified where the minimal data set underlying the results described in your manuscript can be found. PLOS defines a study's minimal data set as the underlying data used to reach the conclusions drawn in the manuscript and any additional data required to replicate the reported study findings in their entirety. All PLOS journals require that the minimal data set be made fully available. For more information about our data policy, please see* [*http://journals.plos.org/plosone/s/data-availability*](http://journals.plos.org/plosone/s/data-availability)*.*

*Upon re-submitting your revised manuscript, please upload your study’s minimal underlying data set as either Supporting Information files or to a stable, public repository and include the relevant URLs, DOIs, or accession numbers within your revised cover letter. For a list of acceptable repositories, please see* [*http://journals.plos.org/plosone/s/data-availability#loc-recommended-repositories*](http://journals.plos.org/plosone/s/data-availability#loc-recommended-repositories)*. Any potentially identifying patient information must be fully anonymized.*

**A minimal data set has been uploaded in OSF (Open Science Framework) repository accessible at https://osf.io/mwd5n/**

**Responses to Reviewers**

We are grateful for the positive comments and useful suggestions. The answers to the queries are reported below. Moreover modifications to the text, that eventually have been incorporated in the revised version of the manuscript, are in bold characters.

**Reviewer #1.** *The paper presents a high quality of processing in terms of methodology, contents and results. As regards the introduction, it would be appropriate to increase the number of references and the length of the introduction, also introducing the background paragraph to provide a broader overview of the existing scientific literature also through a rapid revision of the same.*

**The introduction of the study has been expanded, as suggested, and references included as well. The added text is in bold character in the revised version of the manuscript.**

*From a methodological point of view, I recommend providing the inclusion and exclusion criteria for recruited subjects more explicit because it would give further added value to this high-quality work.*
As suggested by the reviewer, the following paragraph on inclusion and exclusion criteria was added to the manuscript in the methods section.

**“Patients were included in the study if they met all the following criteria: age ≥ 18 years; objectively confirmed diagnosis of SARS-CoV-2 pneumonia obtained by real-time reverse transcription polymerase chain reaction (RT-PCR) assay on naso-pharyngeal swab and/or bronchoalveolar lavage; thoracic imaging (chest X-ray or CT scan) documenting pneumonia. Since, the observational nature of the study, no exclusion criteria, except for age below 18 years, were considered in the protocol.”**

*The results are well represented both graphically and from a descriptive point of view, however, English should be improved.
The discussion is well structured, but I would suggest to increase the references because they are very few compared to the contents reported.*

**As suggested by the reviewer the references in discussion have been implemented and updated.**

**Reviewer #2:** *The authors aimed to evaluate the incidence of deep vein thrombosis (DVT) of the lower limbs in patients hospitalized with COVID-19 pneumonia in a non-ICU setting according to the different waves of the SARS-CoV-2 pandemic.
The topics is relevant. Overall considered the paper is well written but the discussion needs to be a little expanded including more detailed pathophysiological considerations between cardiovascular diseases and COVID-19. For example cite and comment the article by Ielapi N et al. Cardiovascular disease as a biomarker for an increased risk of COVID-19 infection and related poor prognosis. Biomark Med. 2020 Jun;14(9):713-716. doi: 10.2217/bmm-2020-0201.*

As suggested, the following paragraph on pathophysiological considerations has been included in discussion.

**It is well known that SARS-CoV-2 through the binding to ACE2 receptor can trigger direct vascular injury [Ielapi N, Licastro Noemi, Provenzano M et al. Cardiovascular disease as a biomarker for an increased risk of COVID-19 infection and related poor prognosis. Biomark Med, 2020; 14(9):713-716. doi: 10.2217/bmm-2020-0201], and may affect signaling pathways, leading to acute cardiovascular injury. Similarly, the acute systemic inflammatory response syndrome induced by SARS-CoV-2 infection, caused by a dysregulated reaction in pro-inflammatory cytokines release and production (known as cytokines storm), determines endothelial damage with cardiovascular consequences, such as venous thrombosis and cardiac injury. However, what is the role of the different SARS-CoV-2 variants and the effects of vaccination in the determination of different vascular injury burden is currently not known.**
